# Oto-facial syndrome and esophageal atresia, intellectual disability and zygomatic anomalies - expanding the phenotypes associated with *EFTUD2* mutations

**DOI:** 10.1186/1750-1172-8-110

**Published:** 2013-07-24

**Authors:** Claudia Voigt, André Mégarbané, Kornelia Neveling, Johanna Christina Czeschik, Beate Albrecht, Bert Callewaert, Florian von Deimling, Andreas Hehr, Marie Falkenberg Smeland, Rainer König, Alma Kuechler, Carlo Marcelis, Maria Puiu, Willie Reardon, Hilde Monica Frostad Riise Stensland, Bernd Schweiger, Marloes Steehouwer, Christopher Teller, Marcel Martin, Sven Rahmann, Ute Hehr, Han G Brunner, Hermann-Josef Lüdecke, Dagmar Wieczorek

**Affiliations:** 1 Institut für Humangenetik, Universitätsklinikum Essen, Universität Duisburg-Essen, Essen, Germany; 2 Unité de Génétique Médicale et laboratoire associé INSERM à l'Unité UMR_S 910, PôleTechnologie Santé, Université Saint-Joseph, Beirut 545, Lebanon; 3 Department of Human Genetics, Radboud University Medical Centre, Nijmegen, The Netherlands; 4 Department of Pediatrics and Genetics, Center for Medical Genetics, Ghent University Hospital, Ghent, Belgium; 5 Sozialpädiatrisches Zentrum Coburg, Coburg, Germany; 6 Zentrum für Humangenetik, Universitätsklinikum Regensburg, Regensburg, Germany; 7 Department of Medical Genetics, Division of Child and Adolescent Health, University Hospital of North Norway, Tromsø, Norway; 8 Humangenetik, Universitätsklinikum Frankfurt, Frankfurt, Germany; 9 Genetica medicala, Universitatea de Medicina si Farmacie, Timisoara, Romania; 10 National Centre for Medical Genetics, Our Lady’s Hospital for Sick Children, Crumlin, Dublin, Ireland; 11 Institut für Diagnostische und Interventionelle Radiologie und Neuroradiologie, Universitätsklinikum Essen, Universität Duisburg-Essen, Essen, Germany; 12 Institut für Humangenetik, Institut für Humangenetik, Universitätsklinikum Ulm, Ulm, Germany; 13 Bioinformatics, Computer Science XI, TU Dortmund, Dortmund, Germany; 14 Abteilung Genominformatik, Institut für Humangenetik, Universität Duisburg-Essen, Essen, Germany

**Keywords:** *EFTUD2*, Mandibulofacial dysostosis type Guion-Almeida (MFDGA), Esophageal atresia (EA), Oto-facial syndrome with midline malformation, Acrofacial dysostosis type Guion-Almeida (AFDGA)

## Abstract

**Background:**

Mutations in *EFTUD2* were proven to cause a very distinct mandibulofacial dysostosis type Guion-Almeida (MFDGA, OMIM #610536). Recently, gross deletions and mutations in *EFTUD2* were determined to cause syndromic esophageal atresia (EA), as well. We set forth to find further conditions caused by mutations in the *EFTUD2* gene (OMIM *603892).

**Methods and results:**

We performed exome sequencing in two familial cases with clinical features overlapping with MFDGA and EA, but which were previously assumed to represent distinct entities, a syndrome with esophageal atresia, hypoplasia of zygomatic complex, microcephaly, cup-shaped ears, congenital heart defect, and intellectual disability in a mother and her two children [AJMG 143A(11):1135-1142, 2007] and a supposedly autosomal recessive oto-facial syndrome with midline malformations in two sisters [AJMG 132(4):398-401, 2005]. While the analysis of our exome data was in progress, a recent publication made *EFTUD2* mutations highly likely in these families. This hypothesis could be confirmed with exome as well as with Sanger sequencing. Also, in three further sporadic patients, clinically overlapping to these two families, *de novo* mutations within *EFTUD2* were identified by Sanger sequencing. Our clinical and molecular workup of the patients discloses a broad phenotypic spectrum, and describes for the first time an instance of germline mosaicism for an *EFTUD2* mutation.

**Conclusions:**

The clinical features of the eight patients described here further broaden the phenotypic spectrum caused by *EFTUD2* mutations or deletions. We here show, that it not only includes mandibulofacial dysostosis type Guion-Almeida, which should be reclassified as an acrofacial dysostosis because of thumb anomalies (present in 12/35 or 34% of patients) and syndromic esophageal atresia [JMG 49(12). 737-746, 2012], but also the two new syndromes, namely oto-facial syndrome with midline malformations published by Mégarbané et al. [AJMG 132(4): 398-401, 2005] and the syndrome published by Wieczorek et al. [AJMG 143A(11): 1135-1142, 2007] The finding of mild phenotypic features in the mother of one family that could have been overlooked and the possibility of germline mosaicism in apparently healthy parents in the other family should be taken into account when counseling such families.

## Background

High-throughput sequencing facilitates discovery of the molecular etiology of rare syndromes. The discovery of the *DHODH* gene being causative for Miller syndrome [1] was the first autosomal recessive condition to be clarified by exome sequencing. Since then, causative genes for many syndromes have been identified using such approaches.

In 2012, the *EFTUD2* gene was found to cause a very distinct condition with phenotypic overlap with Treacher Collins syndrome, the mandibulofacial dysostosis type Guion-Almeida (MFDGA) [2]. This condition was characterized by microcephaly, a characteristic craniofacial appearance with upslanting palpebral fissures, microtia, preauricular and buccal tags and intellectual disability [3,4]. In the paper by Lines et al. [2] only five of twelve patients had anomalies of the thumbs. Because thumb anomalies were reported in additional patients [5,6], it appeared to be one component of the *EFTUD2* phenotypic spectrum, and we suggested to reclassify MFDGA to acrofacial dysostosis type Guion-Almeida (AFDGA) [7].

Although none of the initially published *EFTUD2* mutation carriers presented with esophageal atresia (EA), Gordon et al. [8] reported on eight patients with *EFTUD2* mutations, esophageal atresia and other features of AFDGA.

This clearly demonstrated that the spectrum of the phenotype is wider and more often complicated by internal malformations than previously suspected. Subsequently, *EFTUD2* mutations were found in patients showing hemifacial microsomia with EA or an asymmetric crying face with EA that were previously diagnosed with CHARGE or Feingold syndromes [6].

Here, we broaden the *EFTUD2*-associated phenotype with the identification of mutations in two previously published, apparently novel familial [9,10], and three sporadic previously unpublished patients. We report on a very mild phenotype in one female patient and describe for the first time a family with suspected germline mosaicism.

## Methods

We obtained written informed consent from the families for participation in this study. The study was performed according to the Declaration of Helsinki protocols and was approved by the local institutional review board (ethical votum 12-5089-BO for CRANIRARE and 11-4878-BO for FACE).

### Exome sequencing and data analyses

Exome sequencing was performed on two different platforms. Exome sequencing for patients 1, 2, and 6 was performed on an Illumina HiSeq2000, whereas exome sequencing for patient 4 and 5 was performed on a SOLiD4 platform (Life Technologies, Carlsbad, CA, USA).

For sequencing on the HiSeq 2000, in family 1 (patients 1 and 2) and patient 6, 1.2 μg genomic DNA was fragmented for library preparation by adaptive focused acoustics on a Covaris S220 (Covaris Inc., Woburn, MA, USA) for 60 sec with a duty cycle of 10%, intensity of 5 and cycles per burst of 200. A library was generated on fragmented DNA using the TruSeq Sample Preparation Kit v2 (Illumina, San Diego, CA, USA) following the low-throughput and gel-free method protocols.

Exome enrichment of Library fragments was performed using the NimbleGen Human SeqCap EZ v3.0 KIT following the manufacturer’s protocol and under consideration of the Technical Note “Targeted sequencing with NimbleGen SeqCap EZ Libraries and Illumina TruSeq DNA samples Prep Kit” released by NimbleGen. All samples were analysed on a Bioanalyzer using the Agilent DNA 1000 kit (Agilent Technologies, Inc., Santa Clara, CA, USA) prior to sequencing on an Illumina HiSeq2000 platform using the paired-end sequencing protocol. Data analyses and filtering was performed as described elsewhere [11].

For samples from patients 4 and 5 (family 2), library preparation was started using 3 μg of genomic DNA. Shearing of DNA was performed on a Covaris TM S2 system. Enrichment of the exomes was done according to the manufacturer’s protocol using Agilent’s SureSelect Human All Exon v.2 Kit (50 Mb). Sequencing was performed on a SOLiD4 sequencing platform from Life Technologies. LifeScope software v2.1 from Life Technologies was used to map color space reads along the hg19 reference genome assembly. The DiBayes algorithm, with high-stringency calling, was used for single-nucleotide variant calling. The small Indel tool was used to detect small insertions and deletions. Exome sequencing data were filtered as described previously [12].

### Sanger sequencing

Genomic DNA was extracted from blood samples, buccal smear and urine using DNA extraction Kits (Flexi Gene DNA Kit, Qiagen, Hilden, Germany).

For confirmation of the *EFTUD2* mutations identified by exome sequencing in patients 1 to 6 and for mutation screening in patients 7 and 8 and the 14 mutation negative patients (see Additional file 1: Table S2), amplification and sequence analysis of individual exons and their flanking regions was done essentially as described by Czeschik et al. [11]. The reference sequence for the description of mutations in the cDNA sequence is Ensembl: ENST00000426333/NCBI: NM_004247.3.

### cDNA analysis

The samples were collected in Tempus Blood RNA tubes (Applied Biosystems, Foster City, CA, USA) and RNA was isolated using the Tempus Spin RNA Isolation kit (Applied Biosystems) as recommended by the supplier. One μg of total RNA was used for cDNA synthesis using the Superscript VILO cDNA synthesis kit (Invitrogen, Carlsbad, CA, USA) as recommended by the supplier. The Primer 3 program (http://primer3.ut.ee) was used to design PCR-primers e23F (e23F: 5’-TTCAGTGAAGGACAGCATCG-3’) and e28R (e28R: 5’-TGGGGTAATTGAGCACAACA-3’) (Sigma-Aldrich, St. Louis, MO, USA), and the region covering exons 24–27 of the *EFTUD2* gene was PCR-amplified (expected product size in wild-type transcript, 635 bp) using the JumpStart REDTaqReadyMix PCR Reaction kit (Sigma-Aldrich) with the following touch-down PCR condition: initial denaturation at 95°C (5 min), then 2 cycles of 95°C (20 s), 63°C (20 s), 72°C (20 s), 2 cycles of 95°C (20 s), 61°C (20 s), 72°C (20 s), 2 cycles of 95°C (20 s), 59°C (20 s), 72°C (20 s), and 28 cycles of 95°C (20 s), 57°C (20 s), 72°C (20 s). PCR-products were analysed on a 2% agarose gel (Ultrapure TM Agarose, Invitrogen), purified using the Illustra ExoStar 1-step kit (GE Healthcare Life Sciences, Buckinghamshire, UK) and sequenced using the e23F and e28R primers and the ABI PRISM BigDye Terminator v.3.1 Cycle Sequencing Kit (Applied Biosystems). Fragments were separated on an ABI3130XL Genetic Analyser (Applied Biosystems) and the sequences were analyzed using Sequencher version 5.0 (Ann Arbor, MI, USA).

### Subcloning and sequence analysis

PCR-products were subcloned into the pCR-4 TOPO vector using the TOPO TA Cloning pCR-4-TOPO Vector kit (Invitrogen) as recommended by the supplier. Clones were analysed and selected for further analysis based on the size of the insert, which was determined by direct colony PCR analysis using the M13-20F and M13R primers (Invitrogen) and the JumpStart REDTaqReadyMix PCR Reaction kit (Sigma-Aldrich). PCR-products were analysed on a 2% agarose gel (Ultrapure TM Agarose, Invitrogen), and selected clones were grown overnight in selective medium (LB containing 100 μg/ml ampicillin). Plasmid DNA was isolated using the QIAprep spin Miniprep kit (Qiagen) and approximately 200 ng of plasmid DNA was sequenced and analyzed as above in both directions using the M13-20F and M13R primers.

## Results

The detailed clinical data are summarized in Table 1 and the identified *EFTUD2* mutations in Table 2.

**Table 1:** **Clinical data in patients with *EFTUD2 *mutations**

	**Patient 1**[9]	**Patient 2**[9]	**Patient 3**[9]	**Patient 4**[10]	**Patient 5**[10]	**Patient 6**	**Patient 7**	**Patient 8**	**Lines et al.**[2]**n=12**	**Bernier et al.**[5]**n=1**	**Gordon et al.**[8]**n=12**	**Need et al.**[15]**n=2**	**Luquetti et al.**[9]**n=3**	**Total n=38**
Sex	F	M	F	M	F	M	M	F	5 f/7 m	n.r.	3 f/2 m*	2 m	3 m	13 f/17 m
Consanguinity	-	-	-	+	+	-	-	-	1/12	n.r.	n.r.	n.r.	n.r.	3/38
Development														
ID	IQ 75	Severe	-	Mild	Mild	Moderate	Moderate	Mild	12/12	n.r.	8/9	2/2	2 mild/1 severe	36/38
Age at walking [mo]	16	39	n.r.	30	24	21	36	18	16-60	n.r.	n.r.	n.r.	n.r.	16-60 mo
Age at first words [mo]	16	-	n.r.	36	30	30	24	12	24-30	n.r.	n.r.	n.r.	n.r.	12-36 mo
Epilepsy	-	-	-	-	-	-	+, Gener.	-	5/10	n.r.	1/10	n.r.	2/3	9/31
Pregnancy														
Polyhydramnios	+	+	n.r.	+	+	+	-	+++	n.r.	n.r.	5/10	n.r.	n.r.	11/17
Measurements														
Gestational weeks at birth	41	34	n.r.	40	40	40	38	37	33-42	n.r.	30.5-41	n.r.	n.r.	
Weight	3010/-1.4	2010/-0.4	n.r.	2500/-2.3	4000/1.3	3180/-1.0	3600/0.5	2900/mean	-2.5 – 0.5	n.r.	-2 – 1.5	n.r.	1 - -2	-2.5 – +1.5
Length	51/-0.8	n.r.	n.r.	48/-1.7	n.r.	52/-0.2	49/-0.5	n.r.	n.r.	n.r.	-2 – 0	n.r.	n.r.	-1.7 - mean
OFC	34/-0.6	n.r.	n.r.	32/-2.2	n.r.	34/-1.2	34/-0.4	32.3/-1 SD	-3.5 - -1.75	n.r.	-3 – 0.5	n.r.	1	-2.2 – +0.5
Age at examination [y]	21	8 ^7^/_12_	Adult	8	2.5	7 ^4^/_12_	3,5	4.5	1 – 13 4/12	n.r.	0.5 – adult	2 and 8	n.r.	0.5 y - adult
Height	158/-1.5	128/-2.4	165/-0.2	114.5/-2.6	84.5/-1.91	126/0.1	98/0	100.7/-1.6	-2 – 1 SD	n.r.	-3 – 2	n.r.	-1 - -4 SD	-4 - +1
Weight	63/0.5	25/-2.4	58/-1.0	21/-1.6	11/-1.3	22.5/-0.6	14,6/-0.2	18.2/1	-4 – 0 SD	n.r.	-3 – 3	n.r.		-4 – +3
OFC	51/-3.7	46/-5.9	53.5/-1.0	46/-5.5	43.4/-5.3	47.5/-3.9	45/-3.3	46.6/-3.3	-6 - -3	Microc.	-3 – 1	Microc.	Normal	-5.9 – Normal
Craniofacial dysmorphism														
Facial asymmetry	-	-	+	-	-	-	-	+ (Mild)	n.r.	n.r.	7/10	1/2	3/3	13/23
Hyperplastic supraorbital ridges	-		-	+	+	-	+	-	n.r.	n.r.	n.r.	n.r.	n.r.	3/8
Frontal bossing	-		-	+	+	-	-/Sloping forehead	-	n.r.	n.r.	n.r.	n.r.	0/2	2/4
Upslanting palpebral fissures	+	-	-	+	+	+	-	- Down slanting	n.r.	n.r.	n.r.	n.r.	0/2	4/10
Epibulbar dermoid	-	-	-	-	-	-	-	-	n.r.	n.r.	n.r.	n.r.	1/3	1/11
Microtia/with squared earlobe	+/+	+/+	-	+/+	+/+	+	+/+	+/+	11/11	n.r.	10/12	2/2	3/3	33/37
Preauricular tag	-	-	-	-	-	-	+	-	10/12	n.r.	4/12	n.r.	3/3	18/35
Preauricular pit	-	-	-	-	-	-	+	-	n.r.	n.r.	n.r.	n.r.	n.r.	1/8
A-/hypoplasia of external ear canal	-	+	-	+	-	Narrow	+	+/+	7/10	n.r.	n.r.	n.r.	3/3	15/21
Hearing loss	bil. cond.	bil. comb.	-	bil. cond.	-.	-	+	+, cond.	10/11	n.r.	11/12	2/2	3/3	31/37
Cleft Palate	-	+	Nasal speech	-	+	+	-	-	6/12	n.r.	2/12	‘1/2	0/3	12/37
Reduced mouth opening	+	+	+	n.r.	n.r.	-	+	+	n.r.	n.r.	n.r.	n.r.	n.r.	5/7
Micrognathia	+	+	-	-	+	+	+	+	12/12	n.r.	10/12	n.r.	3/3	31/36
Malformations														
Tracheostomy	-	+	-	-	-	-	-	+	1/12	n.r.	n.r.	n.r.	n.r.	3/20
Esophageal atresia	+	+	-	+	-	+	-	+	n.r.	n.r.	8/12	n.r.	n.r.	13/20
CHD	ASD	VSD	-	n.r.	n.r.	VSD	-	-	7/12	n.r.	3/12	‘1/2	1/3	15/37
Scoliosis	+	-	-	n.r.	n.r.	n.r.	-	-	n.r.	n.r.	n.r.	n.r.	n.r.	1/5
Cleft of zygomatic bone	bil	bil	unil	n.r.	n.r.	bil	n.r	n.r.	n.r.	n.r.	n.r.	n.r.	3/3	7/7
Choanal atresia	-	-	-	-	-	-	-	Stenosis not atresia +	6/12	n.r.	2/12	n.r.	3/3	12/35
Inner/middle ear malformations	n.r.	n.r.	n.r.	Small middle ear cavity, abnormal malleolus	Absence of middle ear pneum., hypopl. malleus and incus	-	n.r	Small middle ear cavity, normal cochlea and semicircular canals	n.r.	n.r.	n..r.	n.r.	3/3	6/7
Anomalies of hands														
Proximally placed/duplicated thumbs	-	-	-	-	-	-	Slightly proximal placement	-	5/9	+	1/12	2/2	2/3	12/35
Clinodactyly V	+	-	-	n.r.	n.r.	-	n.r.	-	n.r.	n.r.	n.r.	n.r.	n.r.	1/5

* Sex of the remaining patients was not indicated in the paper.

Abbreviations: *ID* intellectual disability, *n.r.* not reported, *bil* bilateral.

**Table 2:** **Summary of heterozygous *EFTUD2 *mutations in this report**

	**Family 1**	**Family 2**	**Patient 6**	**Patient 7**	**Patient 8**
	**Patients 1-3**	**Patients 4, 5**			
Genomic position*	chr17:42949813	chr17:42929870	chr17:42957947	chr17:42929931	chr17:42961093
Nucleotide substitution^#^	c.994+1G>C	c.2622dupT	c.594T>G	c.2562-1G>C	c.351-1G>A
Localization	Intron 11	Exon 26	Exon 8	Intron 25	Intron 4
Amino acid substitution	Predicted change:	p.Ile875Tyrfs*10	p.Tyr198*	p.Arg854Argfs*29	Predicted change:
	skiping of exon 11			p.Arg854Argfs*76	Skipping. of exon 5
	p.Ser290Argfs*2			p.Ala823–Gln859del	p.Asp117Glufs*8

*Reference sequence for the genomic position is GRCh37/hg19 as of February 2009, and ^#^reference sequence for the cDNA position is Ensemble: ENST00000426333/NCBI: NM_004247.3.

### Family 1 (patients 1–3)

**Figure 1:**
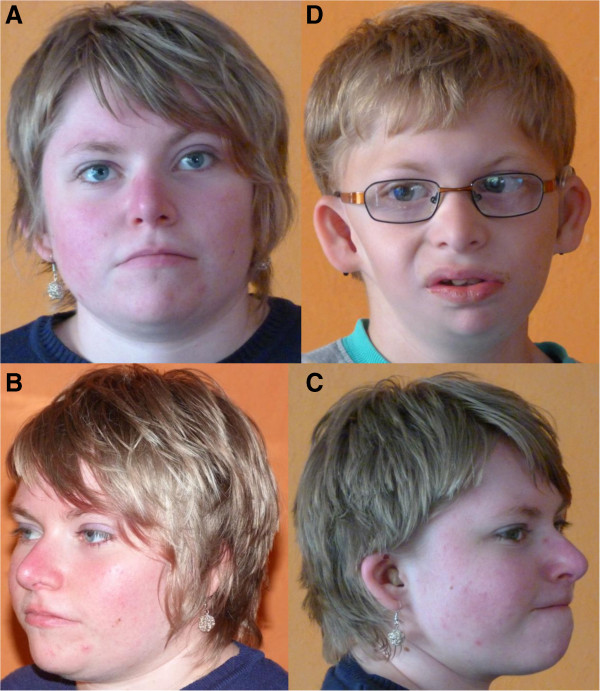
**Current photographs of patients published by Wieczorek et al. **[9]**. (A**, **B**, **C)** Patient 1 at the age of 21 years with upslanting palpebral fissures, downward corners of the mouth and microtia. Micrognathia is not present anymore (no surgical correction performed). **(D)** Patient 2 at the age of 10 ^9^/_12_ years with upslanting palpebral fissures, micrognathia and microtia with dysplastic upper part of the ear and squared earlobe. The patients and their parents gave informed consent to publish the photographs.

This family was published as a new syndrome with esophageal atresia, hypoplasia of the zygomatic complex, microcephaly, cup-shaped ears, congenital heart defect (ASD in patient 1 and VSD in patient 2), and mental retardation [9]. We re-evaluated them in 2012, and the detailed clinical data are depicted in Table 1: At this time, the elder daughter (patient 1) was 21 years old. She had mild intellectual disability (ID). Her body measurements were normal for height and weight [158 cm, -1.5 SD; 63 kg, 0.5 SD], but OFC was still low [51 cm, -3.7 SD]. She had the characteristic facial dysmorphism comprising upslanting palpebral fissures, short philtrum and downturned corners of the mouth (Figure 1A, B). The previously described cup-shaped ears with thickened helices and squared earlobes were still present (Figure 1C).

Her younger brother (patient 2) was more severely affected. He was re-examined at the age of 8 ^7^/_12_ years. Short stature [128 cm, -2.4 SD], low weight [25 kg, -2.4 SD] and microcephaly [46 cm, -5.9 SD] were noted. He suffered from moderate to severe ID. He attended a school for mentally handicapped children, he spoke single words only. As mouth opening was severely restricted, he was still tube fed. The craniofacial phenotype was also more severe in him with upslanting palpebral fissures, small nose with hypoplastic alae nasi, short philtrum and microtia. The upper part of the ear was more severely affected than the lower part with squared earlobes (Figure 1D). The mother (patient 3) appeared intellectually normal. She had a normal head circumference, nasal speech and a scar on her right cheek with underlying hypoplasia of the zygoma. Otherwise she was completely healthy.

**Figure 2:**
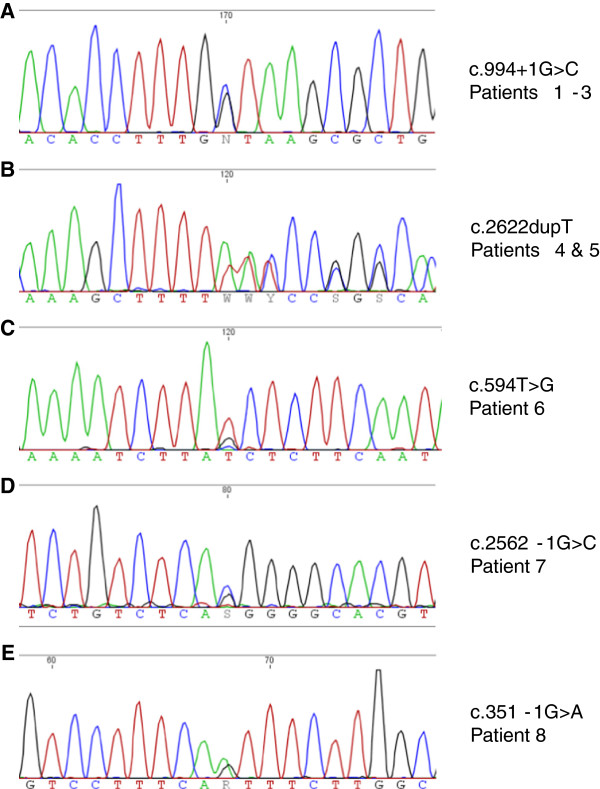
**Electropherograms of *EFTUD2 *mutations. (A)** Family 1. **(B)** Family 2. **(C)** Patient 6. **(D)** Patient 7. **(E)** Patient 8.

To find the molecular basis of this apparently distinct entity, we decided to perform exome sequencing in both affected siblings. During evaluation of our exome data, the paper by Gordon et al. [3] was published, and thus an *EFTUD2* mutation was considered in this family as well. Exome sequencing and confirmation by Sanger sequencing revealed a new splice site mutation c.994+1G>C in both siblings and their more mildly affected mother (Figure 2A). We have not determined the consequence of this splice site mutation experimentally, but the most likely effect is a skipping of exon 11 in the mature mRNA and a shift of the open reading frame that leads to a premature translation stop signal (pSer290Argfs*2). The clinically suspected mosaicism of the mother due to her milder clinical features could not be confirmed in her DNA from blood, saliva and urine, as mutant and wildtype peaks were about the same height as in the blood DNA of her two affected children. We investigated four unaffected family members (two siblings and two further children of patient 3). They were all negative for the splice mutation.

### Family 2 (patients 4 and 5)

**Figure 3:**
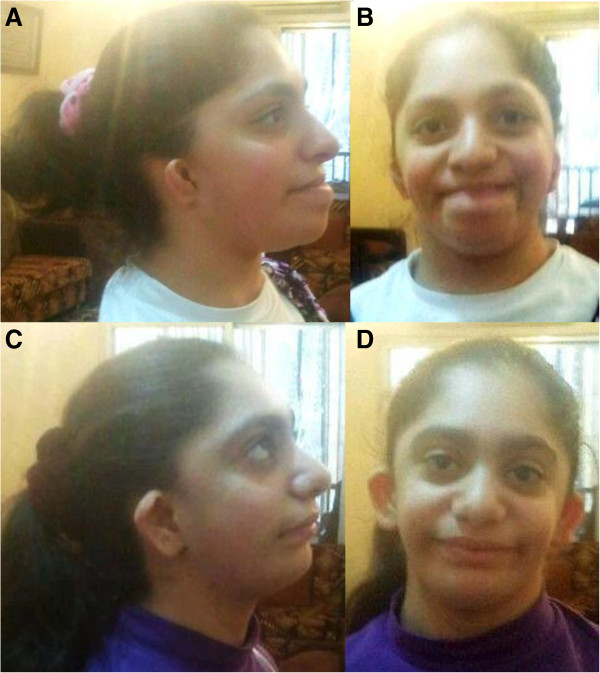
**Update of the craniofacial phenotype of the two sisters with oto-facial syndrome. (A**, **B)** Elder sister at the age of 18 years with receding forehead, large nose and mouth, bilateral microtia with hypoplasia especially of the upper part of the ear with squared earlobe. **(C**, **D)** Younger sister at the age of 12 years with similar, but milder craniofacial dysmorphism.

These two sisters (patients 4 and 5) were published in 2005 as a new autosomal recessive syndrome with midline defects [10]. Both presented with mild ID, microcephaly, microtia with squared earlobes and cleft palate; esophageal atresia was reported in one sister. According to the original publication, they have seven healthy siblings. Unfortunately, only a few current clinical details of the patients are available because the family declined a clinical re-examination: Both affected sisters had moderate ID, the elder one was 18 years old and presented with normal stature [155 cm, -1.6 SD] as well as her younger sister, who was 12 years old [145 cm, -0.8 SD]. The craniofacial phenotype is depicted in Figure 3. The parents gave consent for molecular analyses. Exome sequencing was performed in both sibs to identify the underlying mutation. The previously undescribed heterozygous mutation c.2622dupT, p. Ile875Tyrfs*10 was identified and confirmed by Sanger sequencing in this patient and the affected sibling (Figure 2B). Both parents and six unaffected siblings did not show this mutation. Paternity was confirmed by AmpFLSTR Identifiler kit from Applied Biosystems. Thus, in this family germlinemosaicism in one of the parents was assumed.

### Patient 6

**Figure 4:**
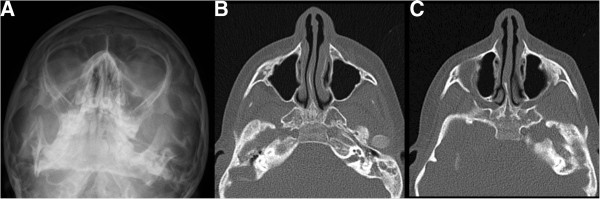
**Zygomatic arch clefting of patient 6.** Occipitomental view of the skull shows only rudimentary development of the zygomatic arch **(A)**. Corresponding computed tomography shows large cleft in the right **(B)** and the left **(C)** zygomatic arch.

**Figure 5:**
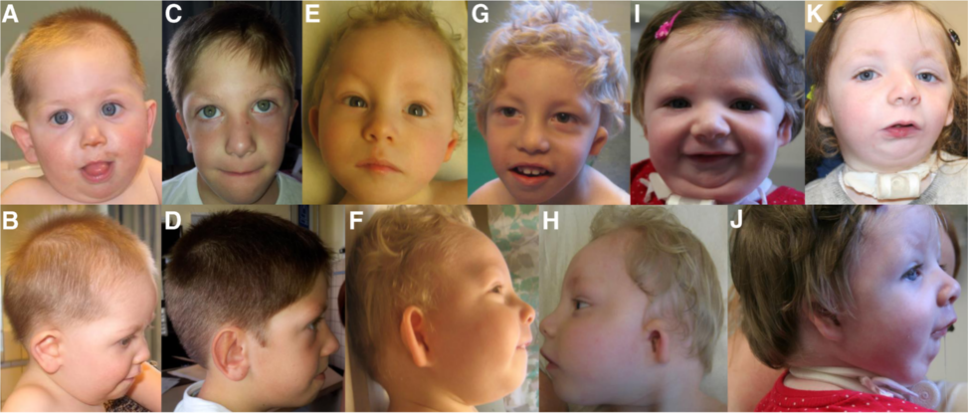
**Craniofacial phenotype of three patients with *de novo EFTUD2 *mutations. (A**, **B)** Patient 6 at the age of 12 months with round face, mildly downslanting palpebral fissures, micrognathia and mild hypoplasia of the upper ear and squared earlobes. **(C**, **D)** Patient 6 at the age of 7 ^4^/_12_ years. Please note that the ears were surgically corrected. **(E**, **F)** Patient 7 at the age of 12 months with normal slant of palpebral fissures, microtia and micrognathia. **(G**, **H)** Patient 7 at the age of 3.5 years with sloping forehead and microtia affecting the upper part of the ear in particular. **(I**, **J)** Patient 8 at the age of 19 months with down-slanting palpebral fissure, microtia with squared earlobes, severe micrognathia and tracheostomy. **(K)** Patient 8 at the age of 4.5 years. Upslanting palpebral fissures and severe micrognathia are still present.

This patient, a third boy in a sibship of three, has not been described before. Pregnancy was complicated by polyhydramnios. He was born at term with normal measurements [weight: 3180 g, -1.0 SD; length: 52 cm, -0.2 SD; OFC: 34 cm, -1.2 SD]. He presented with upslanting palpebral fissures, bilateral microtia with squared earlobe, a branchial tag, cleft soft palate and micrognathia (Figure 4 A,B). Internal malformations consisted of esophageal atresia, ventricular septal defect and bilateral clefts of the zygomatic bones (Figure 2A-C). He walked without support at the age of 21 months and spoke his first words at 30 months. His intellectual disability was mild, brain MRI was normal. Last clinical examination was at age 7 ^4^/_12_ years. He was still microcephalic [OFC: 47.5 cm, -3.9 SD], height and weight were normal [height: 126 cm, 0.1 SD; weight: 22.5 kg, -0.6 SD]. He had an abnormal hair implantation with anterior displacement of the lateral hairline, a round nasal tip, midface retraction, short philtrum and small teeth (Figure 5 C,D). The ears were surgically corrected at the age of 5 years. His limbs were completely normal.

As this patient was very similar to family 1, we performed exome sequencing in this patient parallel to the siblings of family 1. A *de novo* heterozygous *EFTUD2* mutation c.594T>G, p. Tyr198* was identified (Figure 2C) and confirmed by Sanger sequencing. The unaffected parents did not carry the mutation.

### Patient 7

This patient was also previously undescribed. He was the second child of healthy parents. He was born after an uncomplicated pregnancy at 38 weeks of gestation. His birth measurements were normal [weight: 3600 g, -0.5 SD; length: 49 cm, -0.5 SD; OFC: 34 cm, -0.4 SD). At 10 months, his features were microcephaly (41 cm, -3.75 SD], sloping forehead, hyperplastic supraorbital ridges, bilateral microtia with squared earlobes, and auricular fistulas, aplasia of the external ear canal, hearing loss, high arched palate, reduced mouth opening and micrognathia (Figure 5E, F). A preauricular tag was removed in the newborn period. He had no internal malformations. He learned to walk without support at the age of 36 months and spoke his first words at the age of 24 months. He was clinically re-evaluated at the age of 3.5 years (Figure 5G, H). He had normal growth parameters [height: 98 cm, mean; weight: 14.6 kg, -0.2 SD], but microcephaly [OFC: 45 cm, -3.3 SD]. His thumbs were proximally placed, and he did not use a normal ‘thumb-2nd finger grip’. Generalized seizures were present and he was moderately intellectually disabled. He spoke a few words, and used some sign language. Receptive language was far better than spoken language. The tentative diagnosis of a condition caused by an *EFTUD2* mutation was based on clinical grounds and Sanger sequencing was performed. The splice mutation c.2562-1G>C (Figure 2D) was detected in the child and was not present in his unaffected parents. This mutation has not been reported, previously.

**Figure 6:**
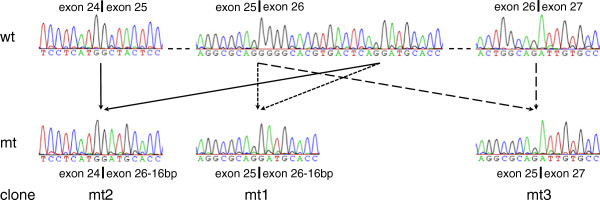
**Expression analysis of the mutant *EFTUD2 *allele of patient 7 with a splice site mutation, c.2562-1G>C.** Part of the *EFTUD2* transcript was amplified, products were subcloned, and individual clones sequenced (see Methods). Four different clones were identified, representing the wild-type allele and three different splice products from the mutant allele. The splice junctions of the wild type (wt, upper row) and of the mutant splice products (mt1, mt2, mt3, lower row) are depicted.

In order to investigate the effect of the splice acceptor site mutation, we analyzed parts of the *EFTUD2* cDNA that was synthesized from RNA isolated from blood leucocytes as described in Methods. Because we obtained more than the two expected PCR products (wild-type, wt, and one mutant, mt), we subcloned the PCR products, and identified clones with four different insert sizes of 635 bp (wt), 619 bp (mt1), 524 bp (mt2) and 481 bp (mt3), respectively. As estimated from the agarose gel analysis of the RT-PCR products (not shown), mt1 appears to represent the most abundant mutant transcript. Sequence analyses of representative clones (Figure 6) revealed that mt1 originated from a mutant transcript that is missing the first 16 bases of exon 26, only, indicating that the exon 26 internal CAG was used as an alternative splice site. The mutant mt2 originated from a transcript that is missing the entire exon 25 plus the first 16 bases of exon 26 (111 bp), and mt3 represents a mutant transcript that lacks the entire exon 26 (154 bp). Whereas mt1 and mt3 would result in shifts of the open reading frame of the respective mRNAs and premature translation stop codons (p.Arg854Argfs*76 and p.Arg854Argfs*29), mt2 should result in an in-frame deletion of 37 amino acids (p.Ala823–Gln859del). These amino acids are highly conserved (100% identity) between many species from puffer fish to mammals, and thus, this internal deletion of amino acids must be considered pathogenic like the frame-shift associated truncations.

### Patient 8

This female was the first child of her non-consanguineous parents, who subsequently had an unaffected daughter. The pregnancy was complicated by polyhydramnios and an amniotic fluid drainage procedure was undertaken at 34 weeks. At birth, she was noted clinically to have severe micrognathia and upper airway obstruction. Detailed examination showed an esophageal atresia, tracheo-esophageal fistula and bilateral choanal atresia, which required the insertion of a tracheostomy, still in situ at age 4.5 years. First examined clinically by a geneticist at day 3 of life, the striking features were the extreme micrognathia and the low set ears, with a “squared off” appearance (Figure 5I, J). The head circumference at birth was normal, 32.7 cm at 37 weeks gestation (−1 SD) but progressively failed to grow, measuring 45 cm at 19 months of age (−1.7 SD), 44.8 cm at 3 years (−3.3 SD) and 46 cm at 4.5 years (−3.3 SD). Ophthalmological assessment was normal, as was cardiac assessment.

Motor development was encouraging. She walked at 18 months and developed several words with the use of a speaking valve. Moreover, she has learned to sign effectively, and that is her main form of communication at age 4.5 years. A psychological assessment at age 4 suggested that language reception was excellent and the problem solving capacity was at or above normal levels. The clinical signs at age 4.5 years remained unchanged, with the ears being small and malformed, especially in the upper helices (Figure 5K), very noteworthy micrognathia, for which surgical advance was planned, and downslanting palpebral fissures. There was slight facial asymmetry.

Investigations had shown normal karyotype and FISH 22q, normal SNP array and normal *TCOF1* mutation analysis. High resolution axial CT scan of the petrous temporal bones showed small ear canals, reduced middle ear cavities, which were fluid filled, and symmetrical but small facial bones. The cochlea and semicircular canal anatomy was essentially normal. Mutation testing of the *EFTUD2* gene showed an intron 4 splice site mutation c.351-1G>A (Figure 2E), which was shown to be *de novo*. We have not determined the consequence of this splice site mutation experimentally but the most likely consequence is a skipping of exon 5 in the mature mRNA and a shift of the open reading frame that leads to a premature translation stop signal (pAsp117Glufs*8).

We sequenced an additional 14 patients with a phenotype overlapping with the previously published patients. Those patients had syndromic esophageal atresia (3/14), microcephaly (8/14), thumb anomalies (1/14), microtia (8/14) = and/or hemifacial microsomia (10/14). However, we could not identify any *EFTUD2* mutation in them. Detailed clinical data are listed in Additional file 1: Table S2 and the craniofacial phenotype is shown in Additional file 1: Figure S1.

## Discussion

The *EFTUD2* (elongation factor Tu GTP-binding domain containing 2) gene encodes U5-116 kD (U5 snRNP-specific protein, 116-KD), a highly conserved spliceosomal GTPase with striking sequence similarity to the ribosomal translation elongation factor EF-2 [13]. Better known is the *S*. *cerevisiae* ortholog of U5-116 kD, the Snu114p. Häcker et al. could show that the Snu114p occupies a central position within the U4/U6-U5 tri-snRNP particle [14]. Fabrizio et al. concluded that the GTP-binding domain of the U5-116 kD protein plays an important role in either the splicing process itself or the recycling of spliceosomal snRNPs [13]. The broad spectrum of clinical anomalies in patients with *EFTUD2* mutations is in agreement with the general cellular function of U5-116 kD protein.

To the best of our knowledge, only six reports on *EFTUD2* mutations/deletions including this report have been published [2,5,6,8,15]. Thirty-one different mutations and four deletions comprising part of or the entire *EFTUD2* gene in a total of 35 non-related patients have been reported so far. The mutations are spread throughout the entire gene and comprise seven missense mutations, eight splice site mutations, seven nonsense mutations and seven frameshift mutations (Additional file 1: Table S2). For two intronic mutations the causality remained elusive [8]. There is no recurrent mutation. Haploinsufficiency is the assumed pathogenic mechanism resulting from these mutations [2].

We analyzed whether there is a correlation between the severity of clinical findings and the type or location of the mutation within the gene by adding characteristic clinical findings of all published patients to Additional file 1: Table S1. One can conclude that there is no obvious correlation, which makes it impossible to predict the phenotypic outcome in mutation carriers. All but three out of 35 individuals with *EFTUD2* mutation were the only affected individual in their families. For the first time, we report an unusually mild *EFTUD2* phenotype in the mother of two severely affected siblings – a daughter and a son [1]. In the absence of further reports on similarly mild *EFTUD2* manifestations it is tempting to speculate that her phenotype resulted from somatic and germline mosaicism in critical tissues despite the presence of a seemingly heterozygous mutation in the investigated blood, saliva and urine specimen. However, alternatively she might also define a milder *EFTUD2* spectrum or carry favorable additional gene variants, which in part rescue the usually more deleterious consequences of *EFTUD2* haploinsufficiency.

There was another familial case with *EFTUD2* mutation published by Gordon et al. [8]. In this family, the mother was also more mildly affected than her daughter, but she had typical findings of *EFTUD2* mutation carriers [8]. However, in all but one patient establishment of diagnosis was possible because of the recognizable clinical phenotype.

Polyhydramnios appears to be a prenatal indicator for a more severe phenotype including esophageal atresia and might potentially guide delineation from prenatal Treacher Collins syndrome and further prenatal molecular genetic workup. In addition, if esophageal atresia is prenatally diagnosed one should carefully evaluate the fetus for signs of mandibulo- or acrofacial dysostoses.

In family 2 of this report, which was first clinically described by Mégabarné et al. [10], the parents were healthy and did not carry the mutation of their two affected children. As family testing confirmed paternity, one of the parents very likely has germline mosaicism, indicating that germline mosaicism should be taken into account when counseling families with apparently *de novo EFTUD2* mutation in one of the children.

## Conclusions

The phenotype in patients with *EFTUD2* mutations is much broader than previously anticipated. We suggest renaming the phenotype Mandibulofacial Dysostosis, type Guion-Almeida to Acrofacial Dysostosis Guion-Almeida (AFDGA), because 12 of 35 patients in this study had thumb anomalies. In addition to AFD type Guion-Almeida and syndromic esophageal atresia, oto-facial syndrome also belongs to the *EFTUD2* mutation spectrum. The clinical phenotype can be very mild as reported in the mother documented by Wieczorek et al. [9]. For the first time preliminary evidence for germline mosaicism is presented in a family published by Mégarbarné et al. [10], which has important implications for genetic counseling.

## Consent

Written informed consent was obtained from the patients themselves/the patient’s parent for the publication of this report and any accompanying images.

### Availability of supporting data

The data set supporting the results of this article is included within the article and the Additional file 1.

## Abbreviations

AFDGA: Acrofacialdysostosis type Guion-Almeida; CT: Computer tomography; DHODH: Dihydroorotate dehydrogenase; EFTUD2: Elongation Factor Tu GTP-binding domain containing 2; EA: Esophageal atresia; GTP: Guanosine triphosphate; KD: Kilodalton; MCA: Multiple congenital anomalies; MFDGA: Mandibulofacialdysostosis type Guion-Almeida; MRI: Magnetic resonance imaging; OFC: Occipitofrontal circumference; SD: Standard deviation; SF3B4: Splicing factor 3B subunit 4; snRNP: Small nuclear ribonucleoproteinparticle; TCOF1: Treacher Collins-Franceschetti syndrome 1; CHD: Congenital heart defect; ID: Intellectual disability.

## Competing interests

The authors declare that they have no competing interests.

## Authors’ contributions

DW, UH and H-JL were involved in design, acquisition and analysis of data, and drafting of the manuscript. AM and HGB were involved in design, acquisition and analysis of data, and made contributions to the draft of the manuscript. CV, AH and HMFRS performed the molecular analyses. JCC, BA, BC, FvD, MFS, RK, AK, CM, MP, WR, BS, MS and CT were involved in acquisition and analysis of data, and made contributions to the draft of the manuscript. MM, KN and SR were involved in analysis of the exome data and made contributions to the draft of the manuscript. All authors read and approved the final manuscript.

## Supplementary Material

Additional file 1 **Oto-facial syndrome and esophageal atresia, intellectual disability and zygomatic anomalies - expanding the phenotypes associated with *EFTUD2 *mutations. Figure S1.** Craniofacial phenotype of patients without *EFTUD2* mutation. A. Patient 13 with mild right-sided hemifacial microsomia. B-D-Patient 17 with bilateral cleft lip/palate, right-sided microtia and left-sided preauricular tags. E, F. Patient 21 with right-sided hemifacial microsomia and microtia at both sides. G, H. Patient 15 with left-sided hemifacial microsomia and left-sided mirror ear. **Table S1.** All reported *EFTUD2* mutations organized to their location within the gene and the associated clinical findings. **Table S2.** Clinical data of 14 patients tested negative for *EFTUD2* mutations. Click here for file
