# Supplementary material for: Oto-facial syndrome and esophageal atresia, intellectual disability and zygomatic anomalies - expanding the phenotypes associated with EFTUD2 mutations
Source: Orphanet J Rare Dis. 2013 Jul 24;8:110. doi: 10.1186/1750-1172-8-110 (PMC3727992; doi:10.1186/1750-1172-8-110)
Supplement: Additional file 1 — Oto-facial syndrome and esophageal atresia, intellectual disability and zygomatic anomalies - expanding the phenotypes associated with EFTUD2 mutations. Figure S1. Craniofacial phenotype of patients without EFTUD2 mutation. A. Patient 13 with mild right-sided hemifacial microsomia. B-D-Patient 17 with bilateral cleft lip/palate, right-sided microtia and left-sided preauricular tags. E, F. Patient 21 with right-sided hemifacial microsomia and microtia at both sides. G, H. Patient 15 with left-sided hemifacial microsomia and left-sided mirror ear. Table S1. All reported EFTUD2 mutations organized to their location within the gene and the associated clinical findings. Table S2. Clinical data of 14 patients tested negative for EFTUD2 mutations. [file 1750-1172-8-110-S1.pdf]

Supplemental Material

Oto-facial syndrome and esophageal atresia, intellectual disability and zygomatic anomalies - expanding the phenotypes associated with *EFTUD2* mutations

Supplementary Figure 1:

Craniofacial phenotype of patients without *EFTUD2* mutation. A. Patient 13 with mild right-sided hemifacial microsomia. B-D- Patient 17 with bilateral cleft lip/palate, right-sided microtia and left-sided preauricular tags. E, F. Patient 21 with right-sided hemifacial microsomia and microtia at both sides. G, H. Patient 15 with left-sided hemifacial microsomia and left-sided mirrow-ear.

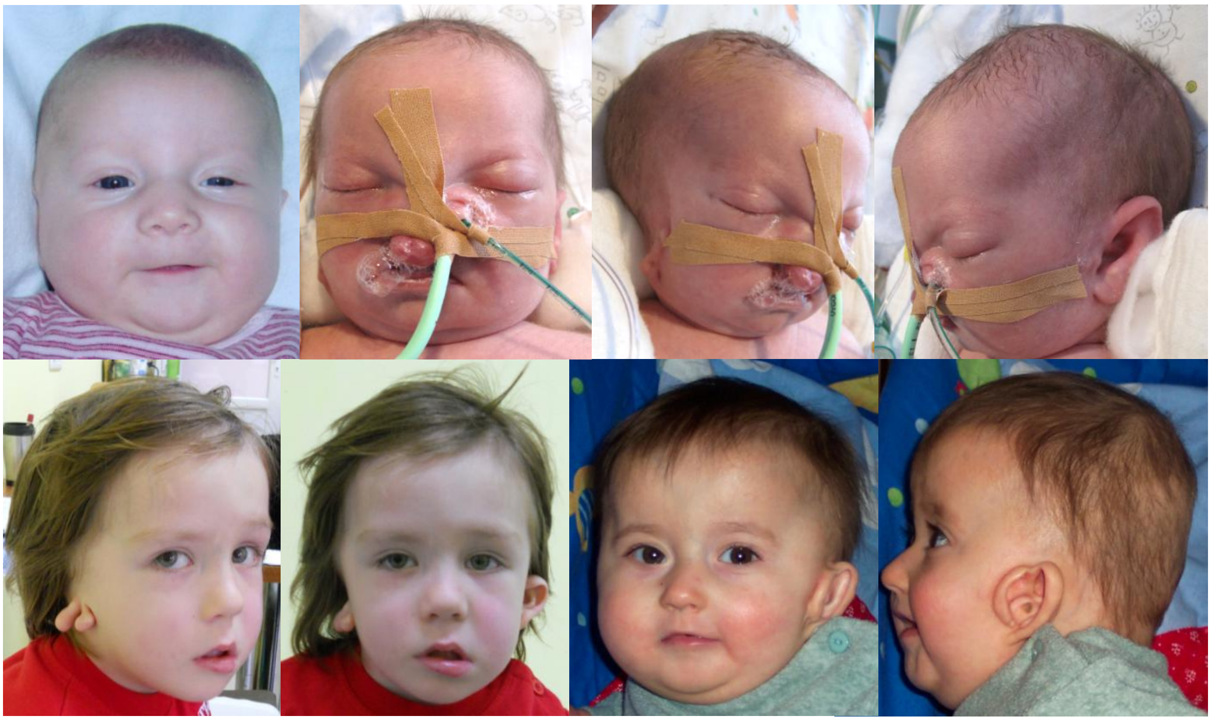

Supplementary Table 1: All reported *EFTUD2* mutations organized to their location within the gene and the associated clinical findings

	Patient / Case [ref]	nucleotide position	amino acid substitution	microcephaly	short stature	ID	thumb anomaly	esophageal atresia	CHD
1	Patient 8 [this report]	c.253-1G>A	splice	+	-	Mild	-	+	-
2	Patient 2 [9]	c.351-1G>T	splice	+	+	mod. to severe	-	-	+
3	Patient 6 [this report]	c.594T>G	p.Tyr198*	+	-	moderate	-	+	+
4	Case 6 [3]	c.619+1G>A	splice	+	-	+	-	+	-
5	Case 7 [3]	c.623A>G	p.His208Arg	+	-	++	-	+	-
6	Case 10 [3]	c.670G>A	p.Gly224Arg	+	-	++	-	-	-
7	Patient 7 [15]	c.698del	p.Glu233Gly*fs**3	+	n.r.	+	-	-	+
8	Patient 2 [5]	c.784C>T	p.Arg262Trp	+	-	mild to moderate	-	-	+
9	Patient 6 [5]	c.784_787deldup788_799TGATCCTGGAGC	p.Arg262*fs**1	+	+	+	+	-	+
10	Patient 1 [15]	c.994+5G>A	splice	+	n.r.	+	+	+	-
11	Patient 1-3 [this report]	c.994+1G>C	splice	+/+/-	-/+/-	mild/severe/-	-/-/-	+/+/-	+/+/-
12	Case 12 [3]	c.1058+3_1058+7del	?	-	-	-	+	+	+
13	Patient 7 [5]	c.1172_1179delGCCTCCCA	p.Ser391Thr*fs**57	+	-	moderate	-	-	+
14	Patient 3 [9]	c.1306C>G	p.Gln436Glu	+	+	Mild	+	-	-
15	Patient 12 [5]	c.1426T>C	p.Cys476Arg	+	-	moderate	+	-	-
16	Patient 10 [5]	c.1607+3A>G	p.Tyr537*fs**25	+	-	mild to moderate	n.r.	-	+
17	Case 5 [3]	c.1705C>T	p.Arg569*	+	-	+	-	+	-
18	Patient 4 [5]	c.1758_1759delTG	p.Ser586Ser*fs**19	+	-	mod. to severe	n.r.	-	-
19	Patient 11 [5]	c.1910T>C	p.Leu637Arg	+	+	mild to moderate	-	-	-
20	Patient 8 [5]	c.2155C>T	p.Gln719*	+	-	moderate	-	-	-
21	Case 8 [3]	c.2198G>A	p.Trp733*	+	-	++	-	-	+
22	Case 3 [3]	c.2259+1G>A	splice	+	+	++	-	+	+
23	Case 9 [3]	c.2347+66A>G	?	-	-	+	-	+	+
24	Patient 1 [9]	c.2485G>A	p.Glu829Lys	-	-	Mild	+	-	-
25	Patient 9 [5]	c.2493C>A	p.Tyr831*	+	-	severe	+	-	+
26	Patient [8]	c.2495C>G	p.Tyr832*	+	n.r.	n.r.	+	-	n.r.
27	Patient 7 [this report]	c.2562-1G>C	splice	+	-	moderate	+	-	-
28	Case 4 [3]	c.2619_2621delinsGGTC	p.Phe874Valfs*11	+	-	++	+	-	-
29	Patient 4,5 [this report]	c.2622dupT	p.Ile875Tyr*fs**10	+/n.r.	-/n.r.	mild/mild	-/-	+/-	n.r./n.r.
30	Patient 3 [5]	c.2770C>T	p.Glu924*	+	-	Mild	+	-	-
31	Case 11 [3]	c.2823+1del	splice	n.r./+	n.r./-	n.r./+	-/-	+/-	-/-
32	Patient 5 [5]	deletion of 10 genes, including *EFTUD2*		+	-	moderate to severe	n.r.	-	+
33	Case 1 [3]	deletion of 4 genes, including *EFTUD2*		-	-	++	-	+	-
34	Case 2 [3]	deletion of 4 genes, including *EFTUD2*		+	n.r.	++	-	+	+
35	Patient 1 [5]	deletion of the last 9 exons of *EFTUD2*		+	-	+	+	-	-
				32/37	6/33	2/37	12/36	15/39	16/36

We changed the mutations from Luquetti et al. according to the described genomic position.

Supplementary Table 2: Clinical data of 14 patients tested negative for *EFTUD2* mutations

	Patient 9	Patient 10	Patient 11	Patient 12	Patient 13	Patient 14	Patient 15	Patient 16	Patient 17	Patient 18	Patient 19	Patient 20	Patient 21	Patient 22
Sex	m	m	m	m	f	m	f	m	f	m	m	m	m	f
Consanguinity	-	-	-	-	-	-	-	-	-	-	n.r.	-	n.r.	-
Development								-	-					
ID	+	- (IQ 99)	+	+	+	-	n.r.	n.r.	n.r.	+	+	+ (sitting with 14 mo.)	+	Mild delay
Age at walking [mo]	13	14	21	n.r.	16	n.r.	n.r.	n.r.	n.r.	15	15	n.r.	12	18
Age at first words [mo]	13	6 - 7	36	n.r.	11	n.r.	n.r.	n.r.	n.r.	24	10	n.r.	9	22
Epilepsy	-	seizures after surgery	-	febrile seizures	-	-	-	-	-	-	+	-	-	-
Pregnancy												ICSI; twin died in 4th wk.		Early rupture of membranes (30w)
Polyhydramnios	-	+	- (oligohydr.)	-	-	-	-	+	+	n.r.	n.r.	-	-	+
Measurements														
Gestational weeks at birth	38	38	32	35	39	40	37	40	37	40	n.r.	42	38	33
Weight/SD	3090/-0.50	2250/-2.43	700/-3.04	1960/-1.50	3340/0.00	3330/-0.67	2390/-1.34	3115/-1.15	2240/-1.69	2400/-2.78	n.r.	3360/-0.94	3100/-0.48	1640/-0.8
Length/SD	51/-0.04	47/-1.78	32/-3.61	40/-2.89	48/-1.40	51/-0.65	46/-1.46	52/-0.22	45/-1.88	49/-1.52	n.r.	52/-0.58	51/-0.04	n.r.
OFC/SD	33/-1.36	33.5/-1.00	23.6/-3.58	30/-1.94	33.5/-0.85	36.5/-0.69	32/-1.35	n.r.	32/-1.36	34/-1.23	n.r.	35/-0.79	n.r.	28.3/-1.3
Age at examination [y]	12 10/12	5 6/12	6	2 1/12	2 1/12	0 9/12	0 6/12	n.r.	0 1/12	5 6/12	3 3/12	1 2/12	3 5/12	4y
Height/SD	140/-1.98	102/-3.16	107/-2.13	85/-0.78	84/-1.06	72/-0.21	64/-1.01	n.r.	50/-1.74	108/-1.92	101/1.04	80/1.60	95/-0.55	88/-4.0
Weight/BMI	28/14.29	13.8/13.26	16.5/14.41	9/12.46	12/17.01	9.3/17.04	5.7/13.92	n.r.	3/12	17/14.57	16/15.68	9.8/15.31	15/16.62	11.5/14.8
OFC/SD	48/-4.12	49.5/-2.07	46.7/-4.15	44/-4.00	45/-2.60	46/0.16	39/3.45	n.r.	n.r.	49/-2.44	48/-2.05	45/-1.65	52/0.87	44.5/-4.4
Craniofacial dysmorphism														
Facial asymmetry	-	+	-	-	+	+	+	+	+	+ (anophth. on the left)	+	+	+	-
Hyperplastic supraorbital ridges	-	-	-	-	-	-	-	-	-	-	-	-	-	+
Frontal bossing	-	-	-	+	-	+	+	-	+	-	-	-	-	+
Upslanting palpebral fissures	+	+	-	-	-	-	-	-	- (downsl.)	-	-	+	-	-
Epibulbar	-	-	-	-	-	-	-	-	-	-	-	-	-	-

dermoid														
Microtia/with squared earlobe	-	+/+	-	-	-	+/-	+/-	+/-	+/-	-	-	+/-	+/-	+
Preauricular tag	-	-	-	-	-	-	+	+	-	+	+	+	-	-
Preauricular pit	-	-	-	-	-	-	-	-	-	-	-	-	+	-
A-/hypoplasia of external ear canal	-	-	-	-	-	-	-	n.r.	+	-	n.r.	+	n.r.	+
Hearing loss	-	+ (cond.)	-	-	-	+	n.r.	n.r.	n.r.	-	n.r.	n.r.		+
Cleft Palate	-	+ (median)	-	-	-	-	+	+ (bilateral)	-	-	+	+ (soft palate)	-	-
Reduced mouth opening	+	-	-	-	-	-	-	n.r.	+	-	n.r.	-	n.r.	-
Micrognathia	+	+	+	+	+	+	+	+	+	-	-	+	-	+
Malformations														
Tracheostomy	-	-	-	-	-	-	-	-	+	-	-	-	-	-
Esophageal atresia	-	-	-	-	-	-	-	+	+ (and esophageal-trachel fistula)	-	-	-	-	+
CHD	-	+ (DORV)	-	+ (VSD, PFO)	+ (pulm. stenosis)	+ (PFO)	-	+ (small VSD)	+ (PDA, VSD, dextropositio)	-	n.r.	+ (VSD, PDA)	-	-
Scoliosis	+ (and rigid spine)	-	-	-	-	-	vertebral deformity	n.r.	n.r.	-	-	+ (and Sprengel deformity)	-	-
Cleft of zygomatic bone	-	-	-	-	-	-	-	-	-	-	n.r.	-	-	n.r.
Choanal atresia	-	-	-	-	-	-	-	-	-	-	-	-	-	-
Inner/middle ear malformations	-	n.r.	-	n.r.	n.r.	-	n.r.	n.r.	n.r.	-	n.r.	n.r.	n.r.	n.r.
Anomalies of hands		edema (after birth)						edema (after birth)						
Proximally placed/duplicated thumbs	-	-	hypoplastic thumbs	-	-	-	-	-	-	-	-	-	-	-
Clinodactyly V	+	-	-	-	-	-	-	-	-	-	-	-	-	-
